# The multifaceted role of lemur tyrosine kinase 3 in health and disease

**DOI:** 10.1098/rsob.210218

**Published:** 2021-09-29

**Authors:** Angeliki Ditsiou, Teresa Gagliano, Mark Samuels, Viviana Vella, Christos Tolias, Georgios Giamas

**Affiliations:** ^1^ Department of Biochemistry and Biomedicine, School of Life Sciences, University of Sussex, JMS Building, Falmer, Brighton BN1 9QG, UK; ^2^ Department of Medicine, University of Udine, Piazzale Kolbe 4, Udine 33100, Italy; ^3^ Department of Neurosurgery, Royal Sussex County Hospital, Brighton and Sussex University Hospitals (BSUH) NHS Trust, Millennium Building, Brighton BN2 5BE, UK

**Keywords:** LMTK3, protein kinase, CNS, cancer, small molecule kinase inhibitor

## Abstract

In the last decade, LMTK3 (lemur tyrosine kinase 3) has emerged as an important player in breast cancer, contributing to the advancement of disease and the acquisition of resistance to therapy through a strikingly complex set of mechanisms. Although the knowledge of its physiological function is largely limited to receptor trafficking in neurons, there is mounting evidence that LMTK3 promotes oncogenesis in a wide variety of cancers. Recent studies have broadened our understanding of LMTK3 and demonstrated its importance in numerous signalling pathways, culminating in the identification of a potent and selective LMTK3 inhibitor. Here, we review the roles of LMTK3 in health and disease and discuss how this research may be used to develop novel therapeutics to advance cancer treatment.

## Graphical abstract

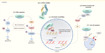

## 1. Introduction

LMTK3 (lemur tyrosine kinase 3) has gained attention in recent years due to the growing evidence of its involvement in a multitude of cancers [1–35]. While initially identified to have a role in ERα (oestrogen receptor alpha) regulation in breast cancer [19], LMTK3 is now known to fuel tumourigenesis through many diverse mechanisms. LMTK3, also known as LMR3 or AATYK3, belongs to the LMTK (LMR or AATYK) family of kinases, along with LMTK1 (also known as LMR1 or AATK) and LMTK2 (also known as LMR2, BREK, KPI2 or AATYK2). This family of kinases performs a wide range of functions in cell signalling and membrane trafficking, while aberrancies in these proteins are linked to diseases such as cystic fibrosis, Alzheimer's disease and cancer [36–40]. Importantly, LMTK3 has been found to be overexpressed in several cancer subtypes, where it contributes to the progression of the disease. LMTK3 mutations have also been identified; however, their clinical significance is yet to be elucidated. About 50% of the identified mutations are missense mutations, while nonsense mutations only occur in 1.71% of the cases (table 1) [41,42].

**Table 1.** Tissue distribution of LMTK3 mutations. Adapted from the Catalogue of Somatic Mutations in Cancer (COSMIC) [41,42]. n.t., not tested; CNS, central nervous system.

tissue	point mutations % mutated	copy number variation variant %	gene expression % regulated	methylation % diff. methylated
adrenal gland	0	0.75	15.19	n.t.
stomach	3.51	0.21	11.93	n.t.
oesophagus	1.24	0.39	9.6	n.t.
pancreas	0.81	n.t	9.5	n.t.
urinary tract	1.87	0.5	6.86	n.t.
lung	1.29	n.t.	6.58	0
thyroid	3.84	n.t.	5.46	n.t.
large intestine	4.07	n.t.	5.41	n.t.
liver	3.02	0.15	5.36	21.72
soft tissue	0.17	0.38	5.32	n.t.
prostate	1.62	n.t.	4.62	2.51
CNS	0.35	0.19	1.15	n.t.
upper aerodigestive tract	2.22	n.t.	4.02	n.t.
cervix	3.37	0.33	3.58	n.t.
breast	1.31	0.34	3.44	0.42
skin	5	n.t.	3.38	n.t.

LMTK3, located at 19q13.33 on chromosome 19, encodes a 1489 amino acid protein (NP_001073903.1) that consists of a kinase domain (aa 162–aa 440), a transmembrane helical segment and a large intrinsically disordered region (IDR), which extends to the end of the C-terminus. Despite its nomenclature, LMTK3 mainly acts as a serine/threonine kinase and phosphorylates various substrates involved in ERα expression and stability, trafficking, gene regulation and oncogenesis as discussed below. The low complexity of the IDR and high homology with other LMTK family members suggest that LMTK3 may possess many unidentified roles beyond those already explored. In addition to its kinase activity, LMTK3 has also been shown to work as a scaffold protein, regulating gene expression. It was described as a ‘randomly created’ scaffold due to the absence of classical scaffolding regions; however, proline-rich domains in the C-terminus may also enable interaction with SH3 domain-containing proteins [43]. The physiological functions of LMTK3 are poorly characterized as most reports focus on LMTK3 aberrancies in different cancers and the predictive and prognostic relevance of LMTK3. Nevertheless, LMTK3 has been studied in normal physiology and proposed to have important trafficking roles in neurons where LMTK3 knockout has a profound impact on the behaviour of mice.

The complex roles played by LMTK3 highlighted the need to solve its crystal structure to support drug discovery efforts. A number of *in silico* approaches have been attempted to model the kinase domain of LMTK3 and subsequently identify potential inhibitors [44–48]; however, the determination of the crystal structure of the kinase domain by our group represents the key milestone in the field [33]. This research ultimately led to the discovery of a potent and selective LMTK3 inhibitor, namely, ‘C28’. This compound slowed cancer growth in xenograft models, demonstrating the therapeutic potential of pharmacological LMTK3 inhibition. Through further structural and functional studies, this significant finding will accelerate the translation of this research into the clinic with great promise to improve cancer therapy.

## 2. The physiological function of LMTK3

One of the more well-characterized physiological functions of LMTK3 is its neurological role. All members of the LMTK family are expressed in the brain, suggesting that they play an important role in the central nervous system (CNS). Kawa *et al*. [49] reported distinct expression patterns between LMTK1, LMTK2 and LMTK3 in the brains of mice. Specifically, LMTK1 was found to be ubiquitously expressed in all regions of the brain, while LMTK2 and LMTK3 were mainly expressed in the cerebral cortex, striatum, cerebellum, hippocampus, olfactory bulb and tubercle. Tomomura *et al*. [50] also highlighted subtle differences in the regional and subcellular localization patterns of different LMTK family members in mouse brains and suggested that they may be involved in the regulation of apoptosis and neurite extension.

Largely consistent with the above studies, LMTK3 was found to be expressed in the thalamus, cerebral cortex and hippocampus of mice, which provided further evidence that LMTK3 is likely to play a role in the CNS [51]. Specifically, this study found that LMTK3 is expressed in layers II to VI of the cerebral motor cortex and areas CA1–3 and dentate gyrus of the hippocampus. Inoue *et al*. [51] attempted to characterize the function of LMTK3 in the CNS *in vivo*. LMTK3^−/−^ mice were compared to wild-type mice and heterozygotes in a number of behavioural tasks. LMTK3^−/−^ mice showed a significant increase in hyperactivity in both novel and acquainted environments compared to their heterozygous and wild-type counterparts. This study also carried out behavioural experiments to assess anxiety. The elevated plus maze is often used in mouse experiments to assess the effectiveness of medications in treating anxiety. Spending more time in the open arm activity and making more visits to the open arms indicates a lower level of anxiety in mice [52]. Inoue *et al*. [51] found that LMTK3^−/−^ mice spent significantly more time in open arms and entered the open arms more frequently than wild-types.

The forced swim test [53] and the tail suspension assay [54] are used to study depression-like behaviour in rodents. In both behavioural assays, the LMTK3^−/−^ mice displayed hyperlocomotion when compared to the wild-types, and it was therefore suggested that the LMTK3^−/−^ mice had less depression-like behaviour [51]. However, as the knockout mice exhibited a hyperactive phenotype, these assays may not be a reliable measure of depression since hyperactivity/attention deficit hyperactivity disorder (ADHD) could be the reason for the observed differences as ADHD has features of hyperactivity, impulsiveness and inattention [55,56].

Dopamine is also suggested to play a role in ADHD-like behaviour [57]. Dopamine is a neurotransmitter which, among others, controls reward, cognition, movement and mood [58]. The dopamine transporter (DAT) reuptakes synaptic dopamine thereby reducing extracellular dopamine levels [59]. DAT^−/−^ mice are used as a model for ADHD. DAT^−/−^ mice have higher levels of extracellular dopamine and display a unique phenotype primarily characterized by hyperlocomotion [60]. Methylphenidate, a licensed medication for the treatment of ADHD, increases synaptic levels of dopamine through reuptake inhibition [61]. Paradoxically, methylphenidate has a calming effect on DAT^−/−^ mice [62]. LMTK3^−/−^ mice had increased levels of striatal dopamine metabolism, suggesting a possible explanation for their hyperlocomotion; however, when LMTK3^−/−^ mice were treated with methylphenidate, they still exhibited a more hyperactive phenotype than their wild-type counterparts [51]. These data suggest that the pathophysiology of hyperlocomotion in LMTK3^−/−^ differs from ADHD and the DAT^−/−^ model.

The *N*-methyl-d-aspartate receptor (NMDA-R) is involved in neuronal plasticity and excitotoxicity [63]. LMTK3-deficient cultured neurons have been shown to have increased levels of intracellular GluN1 and GluN2B subunits of the NMDA-R but the total and surface levels of the receptors were not significantly different to the control [51]. Inoue *et al*. [51] suggested that LMTK3 has a role in the trafficking of the NMDA-R and that the total number of surface receptors is the same because of homeostatic mechanisms, which detect the level of receptor expression on the cell surface, as is the case for AMPA (α-amino-3-hydroxy-5-methyl-4-isoxazolepropionic acid) receptors [64].

The NMDA-R is also implicated in psychiatric conditions such as mood disorders and schizophrenia [65–67]. A recent study looked at the role of LMTK3 in psychiatric conditions. In this follow-up work, further behavioural experiments of the LMTK3^−/−^ mice were carried out and it was suggested that the knockout mice exhibited behavioural characteristics associated with schizophrenia and bipolar disorder [68]. Specifically, the knockout mice had cognitive defects, loss of novelty preference, deficits in prepulse inhibition and hyper-sociability. Lower sociability [69] and depression [70] are frequent clinical findings in patients with schizophrenia. However, in the initial behavioural experiments described above, the LMTK3^−/−^ mice showed a phenotype of reduced depression [51]. Therefore, this evidence suggests that the LMTK3^−/−^ mice could be exhibiting a behavioural pattern consistent with overlapping bipolar and schizophrenia-like behaviour [68]. Schizoaffective-like behaviour has a mixture of both mood and psychotic symptoms [71], and the LMTK3^−/−^ mice could resemble this disease.

Clozapine is an antipsychotic used for treating schizophrenia. When LMTK3^−/−^ mice were treated with acute doses of clozapine, they showed improvement in learning and memory [68]. Moreover, the same study also demonstrated that while glutamate A1 (GluA1) expression was reduced in the forebrain of the knockouts, the levels of GluA1 were increased when treated with clozapine. Electrophysiological experiments also revealed impairment of long-term potentiation in the knockout mice and reduced GluA1 trafficking post-AMPA stimulation [68]. These studies suggest an important role for LMTK3 in trafficking, in line with the functions of other LMTK family members; however, more research is needed into the specific role played by LMTK3.

The potassium chloride co-transporter (KCC2) also plays an important role in neurons through the extrusion of Cl^−^ to maintain a low intracellular chloride concentration, enabling GABA_A_ (type-A γ-aminobutyric acid) receptor- and glycine receptor-mediated post-synaptic inhibition. Potentiation of KCC2 has been proposed as a strategy to treat both epilepsy and neuropathic pain due to its role in counteracting the pathological hyperexcitability of neurons in these disease states [72]. Using immunoprecipitation of KCC2 complexes, followed by blue native polyacrylamide gel electrophoresis (BN-PAGE) coupled with LC-MS/MS, Smalley *et al*. [73] identified three stable protein complexes containing KCC2, isolated from the mouse forebrain. LMTK3 was detected in the largest of these complexes. The phosphorylation sites of KCC2 were also mapped, revealing 11 phosphosites, one of which matched the consensus sequence for LMTK3 (ESRGS^940^) that is described below. Although the function of LMTK3 in this complex was not determined, it may represent an important additional role of LMTK3 in the CNS.

Overall, given the widespread expression of LMTK3 in the brain, it is likely to play a crucial role in the CNS. To date, studies on LMTK3 and the CNS are very limited and the exact physiological role of LMTK3 in the CNS remains unknown, although LMTK3 is proposed to be involved in GluA1 trafficking. LMTK3^−/−^ mice might be a valuable model in trying to both understand the pathophysiology of schizoaffective disorders both at the neuronal network level but also to study the effect of novel antipsychotic medications. Future experiments are needed at a molecular level to understand the exact mechanism through which LMTK3 regulates GluA1. Finally, the comparison of LMTK3^−/−^ mice to wild-types through *in vivo* two-photon imaging will be invaluable in further deciphering the specific roles of LMTK3 in the CNS.

## 3. The intricate role of LMTK3 in cancer

LMTK3 has been implicated in a broad range of cancers, both as a key component of a variety of oncogenic pathways and as a useful predictive and prognostic biomarker (table 2). Although much of the research performed to date has shown LMTK3 to be an oncogene in breast cancer, as extensively described below, LMTK3 was initially found to be linked to leukaemia. In 2009, Tyner *et al*. [1] identified LMTK3 as a potential target in chronic neutrophilic leukaemia (CNL) since patient-derived CNL cells carrying the JAK2 V617F mutation were sensitive to LMTK3 silencing. A previous tyrosine kinome siRNA study in acute myeloid leukaemia (AML) did not identify LMTK3 as a target, highlighting the heterogeneity of cancers and the importance of identifying relevant populations for specific therapies [76]. Nevertheless, LMTK3 may be a useful target to explore in blood cancers.

**Table 2.** Identification and characterization of LMTK3 in various types of cancer (in alphabetical order). CNL, chronic neutrophilic leukaemia; CRC, colorectal cancer; GBM, glioblastoma multiforme; GIST, gastrointestinal stromal tumour; NB, neuroblastoma; NSCLC, non-small-cell lung cancer; PDAC, pancreatic ductal adenocarcinoma.

cancer type	references
bladder	[14]
breast	[16–27,29–35,74]
CNL	[1]
CRC	[11–13]
gastric	[9,10]
GBM	[2]
GIST	[8]
melanoma	[8]
NB	[3]
NSCLC	[4–6]
PDAC	[28]
prostate	[15,35,75]
thyroid	[7]

Several studies have also highlighted a role for LMTK3 in brain tumours. Fazi *et al*. [2] investigated the miRNome and transcriptome of glioblastoma (GBM) tissues and peritumoural regions with the aim of identifying molecular pathways involved in overall survival. LMTK3 was among the possible targets the authors found. Evaluation of the data at the mRNA and protein levels confirmed that LMTK3 is overexpressed in tumoural tissues and peritumoural regions compared to healthy white matter [2]. Somatic mutation of LMTK3 has been identified in neuroblastoma patients; however, the clinical significance of these mutations was not investigated [3].

LMTK3 has been identified as a contributor to both the development and progression of lung cancer. As ERα also has prognostic value in lung cancers, studies have explored LMTK3 as a potential lung cancer biomarker [77]. Higher levels of serum LMTK3 were found in non-small-cell lung cancer (NSCLC) patients, compared to healthy individuals and those with benign lung lesions [4]. Similar results were obtained by Zhang *et al*. [5] in 2015 where it was additionally shown that NSCLC tissue had a higher abundance of LMTK3 compared to healthy lung tissue. Furthermore, LMTK3 has been reported to have a role as a putative transformation suppressor in NSCLC as LMTK3 shRNA resulted in increased anchorage-independent growth as measured by the soft agar colony formation assay [6]. Although the hit was not validated, it may suggest that LMTK3 plays a tumour suppressor role in the early development of NSCLC before taking on an oncogenic role in more advanced disease. These studies emphasize the relevance of LMTK3 as both a potential target and a biomarker in cancer; however, more research is needed into the specific role played by LMTK3 and the balance between its oncogenic and transformation-suppressing functions.

A role of LMTK3 in thyroid malignancies was also described by Lu *et al*. [7]. ERα is overexpressed in thyroid cancers and oestrogen antagonists have been proposed as a treatment [78]; therefore, LMTK3, as a modulator of ERα, may represent a useful target in thyroid carcinomas. The authors found that serum LMTK3 was more abundant in patients with more advanced disease. In addition, tissue samples had a higher abundance of LMTK3 mRNA and protein in malignant thyroid tumours compared to benign lesions. Using the SW579 squamous cell thyroid carcinoma cell line, they found that LMTK3 silencing decreased invasiveness and promoted apoptosis. This study suggests that LMTK3 may play an important role in promoting invasion and cell survival in thyroid cancer [7].

The role of LMTK3 has been widely investigated in gastrointestinal stromal tumours (GISTs). KIT is a receptor tyrosine kinase activated by the ligand stem cell factor (SCF). Mutant KIT is a key driver of GIST growth; therefore, the inhibition of KIT by imatinib is effective in improving patient survival; however, imatinib resistance is a major problem in GIST treatment. Using a human kinome siRNA screen, LMTK3 was identified to have a role as a novel KIT regulator in KIT-mutant GIST and melanoma cells. Klug *et al*. found that LMTK3 regulates the translation of KIT. Loss of LMTK3 in GIST resulted in a decrease in total KIT and a reduction in its downstream signalling, promoting cell death in the GIST430 line [8]. In 2013, Wakatsuki *et al*. [9] found that in a Japanese male cohort of gastric cancer patients, the LMTK3 polymorphism rs9989661 (T > C) T/T genotype was associated with disease-free survival and overall survival. On the other hand, the rs8108419 (G > A) G/G genotype was associated with overall survival in the corresponding female cohort [9]. In another study, in 2014, Li *et al*. [10] demonstrated that LMTK3 is more frequently expressed in gastric cancer tissues compared to normal tissue. In addition, a Kaplan–Meier analysis showed that high levels of LMTK3 are a negative prognostic marker in gastric cancer [10].

Colorectal cancer (CRC) is one of the leading causes of cancer death worldwide [79]. Activation of Wnt/β-catenin signalling is a key driver of CRC and therefore an attractive target for novel therapies. Naik *et al*. [11] used a kinome-wide siRNA screen in human cells stably expressing a luciferase-based Wnt reporter in the presence of Wnt3a to identify novel modulators of the Wnt pathway [11]. The loss of LMTK3 was shown to reduce β-catenin-regulated gene expression in both the primary siRNA screen and an additional shRNA screen, suggesting that LMTK3 plays an important role in Wnt signalling and CRC. Similarly, in 2013 Shi *et al*. [12] found that high serum LMTK3 positively correlates with tumour invasiveness and tumour node metastasis (TNM) stage. The same research group in 2014 also discovered that cellular LMTK3 levels positively correlate with CRC malignancies [13]. The authors found that about 90% of CRC cells stained positive for LMTK3.

In bladder cancer cells, LMTK3 overexpression is positively correlated with cancer progression and worse overall survival. In 2020, Jiang *et al*. demonstrated that silencing of LMTK3 induces G2/M arrest, decreased cell growth and increased apoptosis. Transwell assays also revealed that LMTK3 knockdown reduces invasiveness. They showed that MEK (mitogen-activated protein kinase kinase, MAPKK) and ERK1/2 (extracellular signal-regulated kinase 1/2) phosphorylation levels decrease upon LMTK3 siRNA treatment. In a rescue experiment, the MAPK (mitogen-activated protein kinase) inhibitor U0126 partially rescued the invasive, highly proliferative phenotype of LMTK3-overexpressing cells, indicating that driving MAPK activation is one pathway through which LMTK3 achieves its oncogenic effects [14].

By contrast to the many oncogenic roles of LMTK3, an unexpected function has been described in prostate cancer. During the carcinogenesis of prostate cells, LMTK3 expression is frequently downregulated at both the mRNA and protein level, as opposed to the upregulation seen in many other cancers [15]. Overexpression of LMTK3 in prostate cancer cells has been shown to reduce migration and invasion and induce apoptosis in prostate cancer cell lines. Interestingly, LMTK3 siRNA had no impact on cell viability. Additionally, when injected into nude mice, LMTK3-overexpressing prostate cancer cells formed smaller tumours than wild-type cells. The major driver of this was found to be apoptosis-related as BAX (Bcl-2 associated X-protein) and Caspase-3 were upregulated in the LMTK3-overexpressing tumour tissue and BCL2 (B-cell lymphoma 2) was downregulated. These results point to a tumour suppressor role for LMTK3 in prostate cancer, contrary to the oncogenic roles seen in most other cancers. The authors also found that p-P38 and p-JNK (c-Jun N-terminal kinase) increased while p-AKT and p-ERK1/2 decreased upon LMTK3 treatment and suggested that the observed changes in MAPK subfamily phosphorylation may be the driver of apoptosis in cells overexpressing LMTK3; however, this was not determined experimentally [15].

In aggregate, the studies above underline the pressing need for a better understanding of the elaborate mechanisms involving LMTK3 in different tumour types.

## 4. The role of LMTK3 in breast cancer and ERα regulation

In 2020, 2.3 million new cases of breast cancer were diagnosed and 684 996 deaths were recorded [79]. An improved understanding of the molecular mechanisms underpinning breast cancer development, progression and therapy resistance is therefore essential to achieve improved outcomes in breast cancer patients. Kinases and kinase-related proteins are among the most investigated targets for the development of new therapies to treat cancer. This is particularly true in breast cancer where the roles of these proteins in both tumour progression and response to treatment have been deeply and widely investigated [16–18].

**Figure 1.**
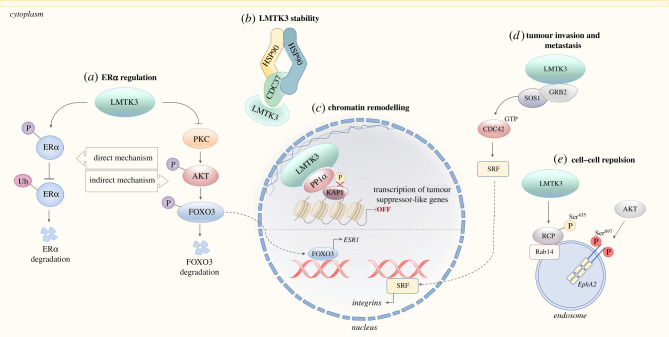
LMTK3 impacts on several facets of tumorigenesis. Graphical summary of the main mechanisms of oncogenic LMTK3 signalling. (*a*) ERα regulation by LMTK3. LMTK3 regulates the activity of ERα at both the mRNA and protein level. LMTK3 reduces the activity of PKC, resulting in decreased levels of phosphorylated AKT and increased binding of FOXO3 to the ESR1 promoter, indirectly increasing transcription of ERα. In addition, LMTK3 directly phosphorylates ERα, promoting stability by protecting it from ubiquitin-mediated proteasomal degradation. Adapted from Johnson & O'Malley [19,80]. (*b*) LMTK3 stability. Like many other oncogenic protein kinases, LMTK3 has recently been identified as an HSP90–CDC37 client protein requiring HSP90 for its folding and stability [33]. (*c*) The chromatin remodelling and transcriptional co-repressor behaviour of LMTK3. LMTK3 binds PP1α and KAP1, promoting KAP1 Ser^824^ dephosphorylation, which results in chromatin condensation. Meanwhile, LMTK3 acts as a scaffolding protein, tethering the heterochromatin complex to the nuclear lamina. In doing so, LMTK3 promotes transcriptional repression of tumour suppressor-like genes. Adapted from Xu *et al*. [29]. (*d*) The role of LMTK3 in proliferation, invasion and migration. LMTK3 increases the abundance of integrin α5β1 by interacting with the adaptor protein GRB2. This interaction recruits SOS1, promoting the activation of Ras and CDC42. This increases the activity of the transcription factor SRF, leading to increased binding at the ITGA5 and ITGB1 promoters, resulting in integrin α5 and β1 upregulation [27]. (*e*) LMTK3 promotes cell–cell repulsion through phosphorylation of RCP. Phosphorylation of RCP by LMTK3 has been shown to be required for the trafficking of EphA2 to the membrane via Rab14-positive endosomes. This phosphorylation event is therefore important in driving tumour dissemination through contributing to cell–cell repulsion [28]. AKT/PKB, protein kinase B; CDC37, cell division cycle 37; CDC42, cell division cycle 42; EphA2, ephrin type-A receptor 2; ERα, oestrogen receptor alpha; ESR1, oestrogen receptor 1; FOXO3, forkhead box O3; GRB2, growth factor receptor-bound protein 2; HSPB1, heat shock protein beta-1; HSP90, heat shock protein 90; ITGA5, integrin subunit alpha 5; ITGB1, integrin subunit beta 1; KAP1, Krüppel-associated box domain-associated protein 1; LMTK3, lemur tyrosine kinase 3; PKC, protein kinase C; PP1α, protein phosphatase 1α; Rab14, Ras-related protein Rab14; RCP, Rab-coupling protein; SOS1, son of sevenless homologue 1; SRF, serum response factor.

In 2011, a landmark study by Giamas *et al*. [19] revealed that LMTK3 is a potent regulator of ERα. A kinome-wide siRNA screen was performed to examine the impact of depletion of different kinases on the expression of the ERα-regulated gene, TFF1 (trefoil factor 1). LMTK3 knockdown strongly reduced the expression of TFF1 in the primary screen, in addition to GREB1 (growth regulation by oestrogen in breast cancer 1) and PGR (progesterone receptor) secondary screens. LMTK3 knockdown also induced a significant reduction in ERα protein levels. Mechanistically, two distinct pathways were found to be responsible. Firstly, LMTK3 affected the stability of ERα through phosphorylation, which protected ERα from ubiquitin-mediated proteasomal degradation (*in vitro* data). In addition, LMTK3 promoted ERα transcription through the inhibition of PKC (protein kinase C), which subsequently reduced the activity of the PKC substrate, AKT through a decrease in Ser^493^ phosphorylation. AKT promotes the degradation of the ESR1 transcriptional activator, FoxO3 (forkhead box O3); therefore, the action of LMTK3 on PKC resulted in the lifting of the inhibition on ESR1 transcription, causing an increase in ERα expression (figure 1). The relevance of this pathway *in vivo* was demonstrated in a xenograft mouse model where LMTK3 siRNA reduced the growth of tumours in mice initiated by ER+ MCF7 cells. Interestingly, LMTK3 was the only regulator of ERα identified to have undergone recent positive Darwinian selection, suggesting an important biological function. Moreover, given the unusual predisposition of humans to ER+ breast cancer, the positive selection of LMTK3 indicates that it could be a key driver of this cancer subtype in humans [19,20].

Clinical analyses also revealed the importance of LMTK3 as a predictive and prognostic marker in breast cancer. In a follow-up study, Stebbing *et al*. [21] found that a high nuclear and cytoplasmic LMTK3 abundance is a strong predictor of more aggressive disease with poorer clinical outcomes. Two intronic polymorphisms were also identified as important markers of tumour recurrence as well as disease-free and overall survival [19]. Tanioka *et al*. [22] detected increased levels of LMTK3 in HER2-positive/ER-positive breast cancer, while overexpression of LMTK3 positively correlated with poor prognosis. These findings were further corroborated by a study by Asano *et al*. [23], supporting the hypothesis that increased LMTK3 expression is an indicator of poor prognosis in ER+ breast cancer.

## 5. The role of LMTK3 in drug resistance

**Figure 2.**
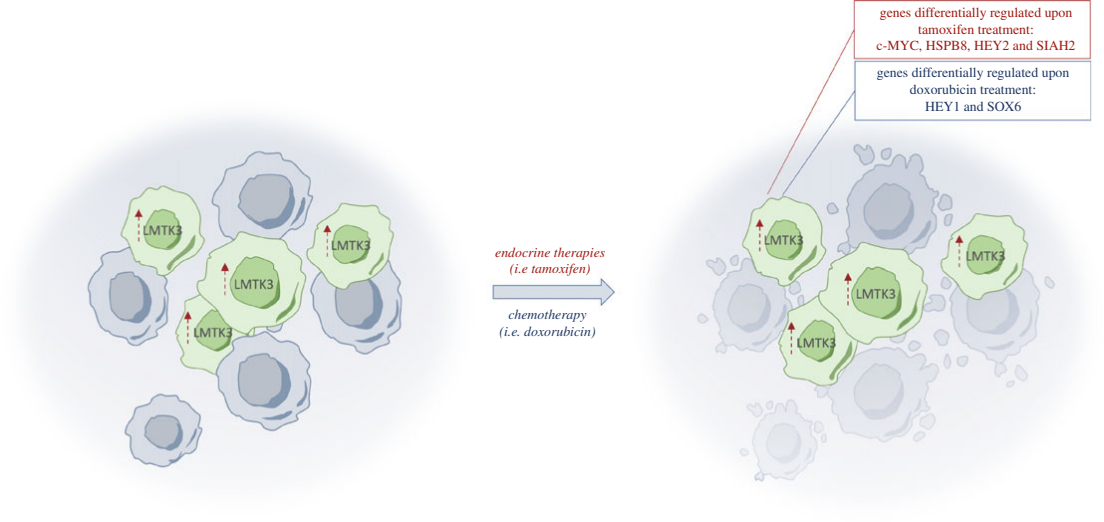
LMTK3-mediated endocrine and cytotoxic drug resistance in breast cancer. An increased LMTK3 abundance confers endocrine and chemotherapy resistance in breast cancer. Interestingly, LMTK3 differentially regulates genes in the presence of tamoxifen or doxorubicin. Upon treatment, cells overexpressing LMTK3 modulate the expression of certain genes in the opposite direction to wild-type cells. For example, in wild-type MCF7 cells, HEY1 is upregulated and SOX6 is downregulated when the cells are treated with doxorubicin; however, this is reversed in MCF7 cells overexpressing LMTK3. LMTK3 also modulates the expression of genes involved in tamoxifen resistance, including c-MYC, HSPB8, HEY2 and SIAH2. c-MYC, proto-oncogene c-MYC; HEY1, hairy/enhancer-of-split related with YRPW motif protein 1; HEY2, hairy/enhancer-of-split related with YRPW motif protein 2; HSPB8, heat shock protein beta-8; LMTK3, lemur tyrosine kinase 3; SIAH2, siah E3 ubiquitin-protein ligase 2; SOX6, SRY-box transcription factor 6.

Shortly after the elucidation of the role of LMTK3 in ERα regulation, Stebbing *et al*. [24] explored the function of LMTK3 in endocrine resistance. The authors found that tamoxifen-resistant BT474 cells could be re-sensitized to tamoxifen in a xenograft mouse model through inhibition of LMTK3. Mechanistically, through a whole-genome microarray analysis, it was demonstrated that LMTK3 silencing affects the expression of a number of genes related to breast cancer progression and importantly, a subset of genes involved in tamoxifen resistance. These included c-MYC (proto-oncogene c-MYC), HEY2 (hairy/enhancer-of-split related with YRPW motif protein 2), SIAH2 (siah E3 ubiquitin-protein ligase 2) and HSPB8 (heat shock protein beta-8). HSPB8 has been shown to reduce autophagy in MCF7 cells, inhibiting cell death in the presence of tamoxifen [81]. LMTK3 was found to promote the translation of HSPB8 and overexpression of LMTK3 reduced tamoxifen-induced cell death in MCF7 cells by increasing HSPB8 abundance. Furthermore, LMTK3 protein levels were found to positively correlate with endocrine resistance where non-responders to aromatase inhibitors (AIs) had a higher abundance of LMTK3 as measured using immunohistochemistry of tumour tissue. Regions of the gene coding for LMTK3 were also found to be amplified or deleted in cfDNA (circulating free DNA) samples taken before and after therapy. Changes in the LMTK3 gene correlated with relapse after tamoxifen therapy. Taken together, these data suggest a role for LMTK3 in both innate and acquired endocrine resistance (figure 2) [24,25]. Cairns *et al*. [32] also showed the involvement of LMTK3 in MIR2052HG-mediated AI resistance, which is thoroughly discussed in a section below (the interplay between non-coding RNAs and LMTK3).

The role of LMTK3 in chemotherapy resistance was further investigated by Stebbing *et al*. [26]. The DNA topoisomerase II inhibitor, doxorubicin, is an effective treatment for breast cancer that works through inducing DSBs (double-strand breaks) in DNA; however, resistance to therapy occurs rapidly and is a major obstacle in breast cancer treatment. DSBs result in γH2AX (phosphorylated histone H2AX) foci formation; however, the formation of these foci was delayed by LMTK3 overexpression [26]. The pattern of ATM Ser^1981^ phosphorylation also differed with a smaller peak in LMTK3-overexpressing cells that appeared more slowly and decayed more rapidly than in wild-type cells. ATM activation is important for the derepression of p21, BAX and PUMA to promote cell cycle control and apoptosis when DSBs are detected. The loss of this response in LMTK3-overexpressing cells reduces the efficacy of chemotherapy drugs reliant on the induction of DNA damage.

Stebbing *et al*. [26] additionally showed that cells overexpressing LMTK3 were less sensitive to doxorubicin as they retained a higher viability and proliferation rate than wild-type cells after treatment. This effect was also consistent *in vivo*, where LMTK3-overexpressing MCF7 cells formed larger subcutaneous tumours than wild-type MCF7 cells in mice undergoing doxorubicin treatment. Transcriptome profiling revealed that over 700 genes are differentially regulated by doxorubicin, depending on LMTK3 expression levels. Many of these are involved in DNA repair and oncogenesis. Notably, HEY1 (hairy/enhancer-of-split related with YRPW motif protein 1) is upregulated and SOX6 (SRY-box transcription factor 6) is downregulated by wild-type cells upon doxorubicin treatment; however, in cells overexpressing LMTK3, this regulation is reversed (figure 2).

In another study, Gao *et al*. [35] sought to identify mediators of IGF1R (insulin-like growth factor 1 receptor) inhibitor resistance, since IGF1R inhibition has been proposed as a strategy to slow cancer growth as it is an important driver of survival and proliferative signalling [82]. Unfortunately, the lack of biomarkers has made it difficult to identify patients that would benefit from IGF1R inhibition; therefore, the authors explored relevant markers of IGF1R inhibitor sensitivity using an siRNA screen. Depletion of LMTK3 was shown to enhance the sensitivity of both prostate and breast cancer cells to an IGF1R inhibitor as demonstrated by a reduction in viability when exposed to an IGF1R tyrosine kinase inhibitor. Since IGF1R promotes proliferation and survival through Ras and LMTK3 is known to activate the Ras pathway, it is likely that LMTK3 is conveying resistance to IGF1R inhibitors by initiating this signalling intracellularly. This demonstrates another avenue through which cancer may become resistant to small molecule inhibitors, via changes in LMTK3 signalling [35].

Overcoming resistance would represent a great progress in breast cancer management and LMTK3 may be a valuable new target. Moreover, LMTK3 could be used for the prediction of treatment responses in order to personalize medicine and identify patients likely to benefit from therapies in order to avoid severe side effects in those unlikely to experience a therapeutic benefit.

## 6. The role of LMTK3 in proliferation, invasion and migration

In addition to conferring drug resistance and regulating ERα, LMTK3 also has roles in promoting invasiveness in breast cancer. Xu *et al*. showed that LMTK3 induces the transcription of the integrin subunits, integrin α_5_ and β_1_. The adaptor protein GRB2 (growth factor receptor-bound protein 2) is an important linker of receptor tyrosine kinases to the Ras signalling pathway. The authors showed that LMTK3 is able to bind directly to GRB2. GRB2 then recruits the guanine nucleotide exchange factor, SOS1 (son of sevenless homologue 1), enabling it to activate both Ras and the Rho GTPase CDC42 (cell division cycle 42). This subsequently promotes the binding of SRF (serum response factor) to the ITGA5 and ITGB1 promoters, resulting in increased transcription (figure 1). The relevance of this pathway was revealed in wound-healing assays and the Boyden chamber assay where LMTK3 knockdown reduced migration, whereas overexpression led to an increase in migration. Additionally, LMTK3 was shown to promote focal adhesion formation. Overexpression of LMTK3 increased motility, as seen by relocalization of FAK (focal adhesion kinase) and vinculin to the leading edges of migrating cells. Adhesion to collagen and fibronectin was also promoted by LMTK3 overexpression, whereas silencing of LMTK3 decreased adhesion. ITGB1 expression is known to be of prognostic significance in breast cancer where overexpression correlates with poor survival [83]. Xu *et al*. [27] finally showed that LMTK3 abundance correlates with ITGB1 expression in breast cancers through immunohistochemistry, suggesting that LMTK3 promotes invasiveness through ITGB1 transcription which reduces survival in breast cancer.

More recently, Gundry *et al*. [28] found that LMTK3 promotes cell–cell repulsion by altering EphA2 (ephrin type-A receptor 2) trafficking. EphA2 is a receptor tyrosine kinase and a driver of cancer cell dissemination. HGF (hepatocyte growth factor) is known to promote invasion and migration in cancer. The authors showed that HGF treatment of cells results in the phosphorylation of RCP (Rab-coupling protein) at Ser^435^ by LMTK3 and EphA2 at Ser^897^ by AKT. These phosphorylations are both required for Rab14 (Ras-related protein Rab14)-mediated recycling of EphA2 to the membrane. Knockout of either RCP or EphA2 in PDAC (pancreatic ductal adenocarcinoma) mice models resulted in a reduction in invasive behaviour. The authors suggested that the rerouting of EphA2 from the constitutive Rab11-mediated fast recycling pathway to the slower Rab14-mediated pathway, by LMTK3 and AKT phosphorylation of RCP and EphA2, respectively, enables cell–cell repulsion through cytoskeletal responses. By spending more time intracellularly, EphA2 was proposed to partake in signalling pathways to promote repulsion; however, more research is needed into the exact mechanism through which this occurs. Overall, the authors demonstrated that LMTK3 is a key component of this trafficking pathway where its kinase activity is essential for the diversion of endosomal cargo to a Rab14-dependent recycling pathway where it drives HGF-mediated cell scattering (figure 1). Interestingly, the RRSS^435^ phosphorylation site of RCP matches the recently elucidated consensus sequence of LMTK3 that is presented below, further confirming this finding.

## 7. LMTK3 as a repressor of tumour suppressor-like genes via chromatin remodelling

In 2015, Xu *et al*. examined the nuclear effects of LMTK3, which revealed an association between LMTK3 and repressive chromatin markers. Interestingly, LMTK3 brought about this effect by working as a scaffold protein to facilitate the interaction of the transcriptional co-repressor, KAP1 (Krüppel-associated box domain-associated protein 1) and a KAP1 phosphatase, PP1α (protein phosphatase 1α) [29]. The authors demonstrated that LMTK3 localizes to chromatin in an ERα-independent manner in both the ER+ cell line, MCF7 and the TNBC line, MDA-MB-231. Immunoprecipitation revealed an interaction between LMTK3 and KAP1. *In vitro* kinase assays, however, did not indicate that LMTK3 phosphorylates KAP1. It was instead shown that the KAP1 phosphatase, PP1α, is an important stabilizer of the LMTK3–KAP1 interaction and that the three proteins colocalize in the nucleus. Further to this, the authors demonstrated that PP1α dephosphorylates LMTK3-bound KAP1 at Ser^824^, which results in transcriptional silencing and chromatin condensation through the trimethylation of Histone H3 lysine 9 (H3K9me3). Moreover, the interaction leads to the tethering of the H3K9me3-marked heterochromatin to the nuclear periphery (figure 1). Overall, LMTK3, KAP1 and PP1α are co-expressed in the majority of breast cancers where they collaborate to repress tumour suppressor-like genes through the remodelling of chromatin, which promotes a more aggressive phenotype.

## 8. The interplay between non-coding RNAs and LMTK3

Besides the numerous protein interactions, LMTK3 also takes part in some interesting pathways involving non-coding RNAs. The miRNAs miR-34a and miR-182 have been shown to target LMTK3 transcripts, reducing the abundance of LMTK3 mRNA and protein. Functionally, this results in a decrease in proliferation, invasion and migration. It was found that LMTK3 reduces the abundance of miR-34a, miR-182 and miR-196-a2 through sequestration of their respective pri-miRNAs, inhibiting further processing. Through doing this, LMTK3 avoids miRNA-mediated downregulation. LMTK3 was shown to bind to DEAD-box RNA helicase p68 (DDX5) and the pri-miRNAs of these three miRNAs, promoting their processing into pre-miRNA but inhibiting further processing into functional miRNAs, thereby protecting itself from downregulation [30,31]. miR-34a is known to have a tumour suppressor role; however, this study further demonstrated that the oncogenic miRNA, miR-182, also has a tumour suppressor role in breast cancer cells overexpressing LMTK3, highlighting the complex signalling network surrounding LMTK3 regulation.

The long non-coding RNA, MIR2052HG, has also been shown to play a role in resistance to endocrine therapy through the upregulation of LMTK3. Ingle *et al*. had previously demonstrated that MIR2052HG and ESR1 transcription are induced by oestrogen and AIs in some SNP genotypes. Further, they observed that MIR2052HG silencing results in a decrease in ERα expression. The increase in ERα abundance when MIR2052HG is overexpressed was demonstrated to be via the AKT/FOXO3 pathway, which increased transcription of ESR1 through inhibition of ERα degradation. As LMTK3 is involved in ERα regulation through both of these pathways, Cairns *et al*. explored the role of MIR2052HG in LMTK3 regulation where they found that MIR2052HG silencing strongly reduces the expression of LMTK3. Moreover, they found that LMTK3 overexpression could rescue the low proliferation rate of cells with downregulated MIR2052HG. MIR2052HG was found to promote LMTK3 transcription through recruitment of EGR1 (early growth response 1) to the LMTK3 promoter, increasing the abundance of ERα through promoting transcription and inhibiting degradation [32]. The authors also hypothesized that LMTK3 upregulation may be responsible for the AI resistance conveyed by MIR2052HG overexpression. They found LMTK3 to be upregulated in cells with wild-type MIR2052HG upon AI treatment; however, LMTK3 was downregulated in cells with the MIR2052HG variant genotypes, rs4476990 and rs3802201 after treatment with AIs. Cells with the variant genotypes were also more sensitive to aromatase inhibition than wild-type cells. In addition, LMTK3 overexpression desensitized cells to AIs. These findings suggest that LMTK3 is upregulated upon treatment with AIs through MIR2052HG and EGR1 where it may contribute to AI resistance through ERα upregulation and stabilization [32]. Overall, these studies emphasize the breadth of the LMTK3 signalling network, highlighting the importance of further research into LMTK3 pathways.

## 9. Interrogating the LMTK3 signalling circuits

To further decipher the downstream pathways of LMTK3, a positional scanning peptide library (PSPL) approach was taken to determine the consensus phosphorylation sequence of LMTK3. Initially, the consensus peptide substrate (LMTK3-tide: WRRFSFCMC) was designed and then radiolabelled *in vitro* kinase assays of the LMTK3-tide with amino acid substitutions were carried out, revealing a strict requirement for arginine (R) residues at positions -3 and/or -2 [33]. The results were corroborated by a PepChip kinase assay, which also highlighted the importance of arginine at positions -3 and/or -2 [33]. Interestingly, as discussed above, this consensus sequence matched those seen in RCP and one of the putative phosphorylation sites in KCC2, further confirming the validity of this approach [28,73]. It is noteworthy that phosphorylation sites beyond the canonical LMTK3 consensus sequence may also be substrates of LMTK3 as is seen with various other kinases.

By employing a quantitative mass spectrometry analysis using stable isotope labelling of amino acids in cell culture (SILAC), precious phosphoproteomic data were also gathered on LMTK3-regulated proteins in MCF7 breast cancer cells. A multitude of direct and indirect targets of LMTK3 were uncovered with many hits having known roles in promoting oncogenesis. HSP27 (heat shock 27 kDa protein) was among the most significant hits, additionally appearing in the Pepchip microarray and *in vitro* kinase assays. HSP27 has been previously implicated in breast cancer progression and therapy resistance; therefore, the phosphorylation at Ser^15^ and Ser^82^ by LMTK3 may indicate an important pathway that requires further elucidation [84].

Importantly, the data collected from these complimentary approaches will help decode the complex signalling pathways surrounding LMTK3, enabling their integration into the wider network of cancer signalling. With the knowledge that LMTK3 is heavily involved in promoting the progression and development of both ER+ and triple-negative breast cancer, further research into candidate substrates will be crucial to develop therapeutics targeting these pathways to improve cancer treatment.

## 10. The translational potential of LMTK3 in cancer

As described above, LMTK3 has been repeatedly demonstrated to partake in oncogenic processes, suggesting that inhibition of LMTK3 may represent a useful therapeutic tool. Although earlier studies have attempted to develop LMTK3 inhibitors, these have lacked *in vivo* and pharmacological data [45–48,74]; therefore, the need for potent and selective LMTK3 inhibitors remained. However, in 2020, by adopting a robust high-throughput screening approach combined with various biochemical, biophysical and cellular assays, C28 was identified as a potent, orally available and highly selective ATP-competitive inhibitor targeting LMTK3. Pre-clinical data demonstrated strong anti-cancer activity in the NCI-60 cell line panel and experiments in xenograft mouse models revealed that the potency and selectivity translated well *in vivo* [33].

**Figure 3.**
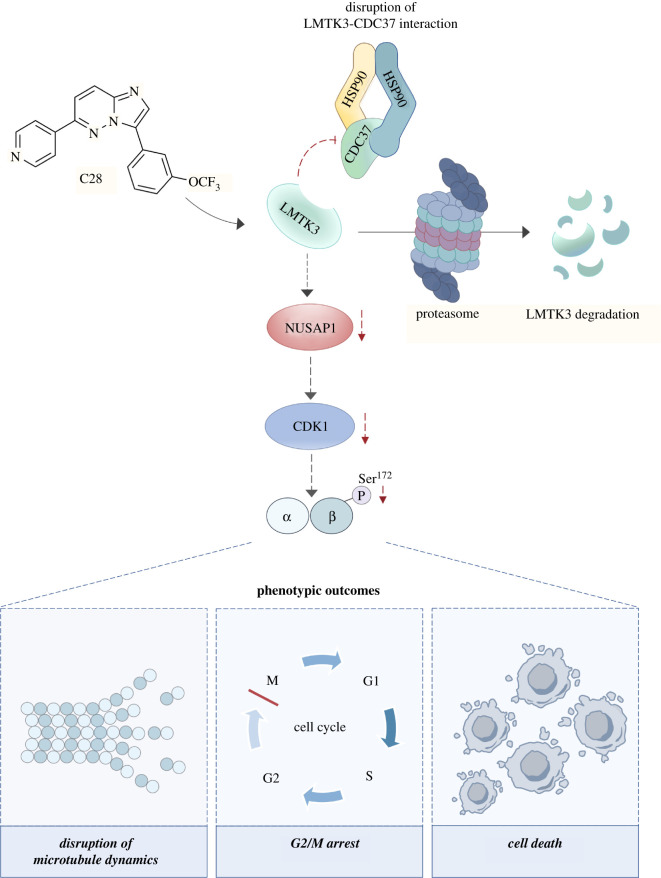
The mechanism of action of C28. LMTK3 has emerged as a critical mediator of oncogenic functions and is therefore considered to be a promising target of anti-cancer therapies. The small molecule C28 was recently identified as a potent and selective LMTK3 inhibitor. C28 acts by inducing the proteasome-mediated degradation of LMTK3 through chaperone deprivation. Furthermore, the depletion of LMTK3 by C28 results in microtubule instability with subsequent G2/M cell cycle arrest and cell death through downregulating NUSAP1 and the NUSAP1-regulated protein CDK1. This also reduces the phosphorylation of βIII tubulin at Ser^172^ [33,34]. CDK1, cyclin-dependent kinase 1; LMTK3, lemur tyrosine kinase 3; NUSAP1, nucleolar and spindle associated protein 1.

Interestingly, while studying the mechanism of action of C28, we discovered that LMTK3 abundance decreases following prolonged treatment of cells with C28. LMTK3 was found to be an HSP90–CDC37 (heat shock protein 90-cell division cycle 37) client protein, which requires HSP90 for folding and stability (figure 1). Experiments revealed that C28 has a dual mechanism of action, both competing for the ATP binding site of LMTK3 and promoting LMTK3 degradation through chaperone deprivation (figure 3). By depriving LMTK3 of HSP90, C28 promotes kinase instability and proteasomal degradation of LMTK3. With oncogenic roles as both a kinase and scaffold protein, this dual mechanism of action will probably provide more therapeutic benefit than a kinase inhibitor alone. Additionally, LMTK3 was found to directly phosphorylate CDC37; therefore, research is needed into the role of LMTK3 on the HSP90–CDC37 chaperone system as a whole [33].

Further characterization of C28 revealed an interesting effect on the cell cycle [34]. In addition to LMTK3 inhibition, C28 was found to induce apoptosis and G2/M arrest in breast cancer cells, indicating that C28 may exert its anti-cancer effects through an additional mechanism. This phenotype reflected that of cells treated with microtubule destabilizing drugs, suggesting that C28 interferes with microtubule dynamics. This was confirmed through immunofluorescence, which showed abnormalities in the mitotic spindle in metaphase cells after treatment with C28. C28 also caused a dose-dependent decrease in insoluble, polymerized tubulin. C28 does not bind directly to tubulin; therefore, a proteomics approach was taken to elucidate the mechanism. Both siRNA against LMTK3 and C28 reduced the abundance of the microtubule-associated protein NUSAP1 (nucleolar and spindle associated protein 1), the NUSAP1-regulated protein CDK1 (cyclin-dependent kinase 1) and decreased the level of phosphorylated βIII tubulin at Ser^172^. The phenotype of cells deficient in NUSAP1 is consistent with that of C28-treated cells. Immunoprecipitation demonstrated that LMTK3 stably interacts with NUSAP1, indicating that the C28-mediated NUSAP1 depletion is likely a result of LMTK3 inhibition and not an off-target effect of C28; however, more research is needed into the exact mechanism (figure 3).

Overall, the substantial volume of functional and mechanistic pre-clinical data presented by these studies, along with the newly solved structure of LMTK3, will aid in optimizing LMTK3 inhibitors with prospective value to cancer patients.

## 11. Concluding remarks

The studies discussed above portray LMTK3 as an exceptionally versatile protein kinase and highlight its complex role both in normal physiology and pathological conditions, most notably cancer. So far we are probably only aware of a small proportion of the LMTK3-mediated roles across different cell types and further investigation is certainly required to unveil the full functional spectrum of LMTK3 in the coming years. Although several groups have examined the physiological role of LMTK3, the majority of studies are principally focused on cancer, while a large percentage of this work is related to clinical studies. Particular emphasis should therefore be placed on mechanistic investigations that will shed more light on the functions of LMTK3. For example, very little is known about the upstream regulation of LMTK3 and elucidating these mechanisms will help define the greater network in which LMTK3 acts. Moreover, optimization of LMTK3 inhibitors on the basis of the work that led to the identification and characterization of C28 presents great translational potential. In summary, research on LMTK3 could have a huge impact on patients, while being beneficial to the scientific community investigating similar fields.

## Data accessibility

This article has no additional data.
